# A case series in patients with enteropathy and granulomatous diseases

**DOI:** 10.1186/s12876-015-0292-4

**Published:** 2015-05-23

**Authors:** Tassilo Kruis, Korinna Jöhrens, Verena Moos, Imke Puls, Britta Siegmund, Severin Daum, Michael Schumann

**Affiliations:** 1 Medical Department I (Gastroenterology, Infectious Diseases, Rheumatology), Charité – Universitätsmedizin Berlin, Campus Benjamin Franklin, Hindenburgdamm 30, Berlin, 12200 Germany; 2 Institute for Pathology, Charité – Universitätsmedizin Berlin, Campus Mitte, Berlin, Germany; 3 Department for Psychiatry, Charité – Universitätsmedizin Berlin, Campus Mitte, Berlin, Germany

**Keywords:** Sarcoidosis, Löfgren’s syndrome, Celiac disease, Autoimmune enteropathy, AIE-75, HLA-DR3/DQ2

## Abstract

**Background:**

Although sarcoidosis and celiac disease are both chronic immunologic disorders involving multiple organ systems, reports about association of diseases in individual patients are sparse. While sarcoidosis is a chronic granulomatous disease presumably reflecting an exaggerated response to an unknown antigen, celiac disease is a T cell-driven disease triggered by ingestion of gluten, a protein composite found in wheat and related grains.

**Case presentation:**

We present three cases with a longstanding history of sarcoidosis that have been additionally diagnosed with celiac-like enteropathy. In two cases, celiac disease was established applying celiac-specific serology and duodenal histology, while one case was revealed as an AIE-75-positive autoimmune enteropathy.

The HLA-DR3/DQ2 haplotype was confirmed in both celiac patients, hence confirming previous data of linkage disequilibrium as a cause for disease association. Remarkably, one celiac patient presented with granulomatous nodulae in the ileum, thus reflecting an intestinal sarcoid manifestation. In contrast the patient with an autoimmune enteropathy, was HLA-DQ9/DQ6-positive, also arguing against CD.

**Conclusions:**

Associations of sarcoidosis and celiac disease are rare but do occur. Determining the HLA status in patients with complex autoimmune associations might help classifying involved disease entities.

## Background

In 1984, Douglas et al. reported on five patients with the combined diagnoses of celiac disease (CD) and sarcoidosis [1]. Since then, other authors provided evidence for an association of these disorders. However, evidence remains limited to a small number of case reports [2, 3] and studies [4–6].

CD is a chronic inflammatory small intestinal disease with a prevalence of 1 % induced by gluten and related storage proteins of wheat, barley, and rye. Its pathogenic keystone is the presentation of processed gliadin peptides by human leukocyte antigen (HLA)-DQ2 and -DQ8 molecules hence triggering an adaptive immune response against various structures of the duodenal mucosa [7]. At the peak of inflammation, the architecture of the small intestinal mucosa is severely altered (i.e. villous atrophy, crypt hyperplasia and increased numbers of intraepithelial T cells) resulting in malabsorption. Most CD patients, who succeed to eliminate gluten from their diet, will achieve regeneration of their duodenal mucosa with subsequent clinical normalization. Population-based twin studies revealed a strong genetic component of CD [8]. These studies were followed by genome-wide association studies elucidating the genetic complexity of CD with HLA class II alleles as major contributing factors [9].

A small percentage of patients with CD will not respond to a gluten-free diet (GFD). In these cases, refractory celiac disease (RCD) should be considered after reviewing stringency of GFD and exclusion of other differential diagnoses. In RCD type I, intraepithelial lymphocytes (IEL) are characterized by normal expression of T-cell antigens as well as polyclonal T-cell receptor gene rearrangement. RCD type I is responsive to immunosuppressive treatment. In contrast, monoclonality of IEL accompanied by loss of T-cell antigens defines RCD type II that is nowadays regarded as an early stage of an enteropathy-associated T cell lymphoma [10].

Sarcoidosis is a multiorgan disease predominantly affecting lung, skin, and eyes with the pathologic hallmark of granuloma formation. Its aetiology is still enigmatic. The current literature suggests an exaggerated immune response to non-degradable pathogen-associated molecular patterns of mycobacteria and propionibacteria but also other organic and inorganic particles. The prevalence of sarcoidosis ranges from 4.7-64:100000 individuals with highest rates in northern Europeans and African-Americans [11].

Common presenting symptoms of sarcoidosis include fatigue, persistent cough, skin lesions, erythema nodosum, and incidental pathologic findings on chest radiograph. Diagnosis should be based on three criteria: (**i**) compatible clinical presentation, (**ii**) evidence of non-caseating epithelioid-cell granulomas, and (**iii**) exclusion of alternative granulomatous disorders [12]. Evidence from genetic association studies suggest that sarcoidosis is a heterogeneous disease with distinct subgroups as particular HLA class II alleles influence the course of disease. While HLA-DR3 predisposes to an acute self-limiting course as seen in Löfgren’s syndrome, HLA-DR14 and -DR15 are associated with chronic disease. Interestingly, it was shown for sarcoidosis that HLA-DR3 is in strong linkage disequilibrium with HLA-DQ2 leading to an equally strong association of the latter to a favourable course [13].

Both CD and sarcoidosis are complex diseases and the establishment of a definite diagnosis can be difficult in individual cases. Here we report on three patients with longstanding courses of autoimmune disease reflected by a malabsorptive syndrome with duodenal villous atrophy and a sarcoid-like granulomatosis. The differential diagnosis in particular with regard to refractory CD will be discussed based on a thorough clinical work-up, including genetic and immunological testing, that aimed to shed light on the pathophysiology of the disease association.

## Consent

Written informed consent was obtained from the patients for publication of the case reports and any accompanying images. A copy of the written consent is available for review by the Editor of this journal.

## Case presentations

For clinical characteristics refer to Table 1.

**Table 1:** Patient characteristics

Patient	A	B	C
Major complaints	Sicca syndrome, arthralgias, chronic diarrhoea, weight loss	Dyspnoea on exertion, chronic diarrhoea, weight loss, gait abnormalities	Chronic diarrhoea since childhood, weight loss, arthralgias
Malabsorptive syndrome			
Age at dx	33 years	34 years	35 years
Duodenal histology	Total villous atrophy	Total villous atrophy, crypt hyperplasia, intraepithelial lymphocytosis	Total villous atrophy, crypt hyperplasia
Serology	Tg-IgA positive	Tg-IgA repeatedly negative, AIE-75 antibodies elevated	Gliadin-IgA/IgG elevated
Response to GFD	Temporary improvement of symptoms, normalization of histologic and serologic findings	No improvement of symptoms or histologic findings	Temporary improvement of symptoms, normalization of histologic and serologic findings
Currently on GFD	Yes	No	Yes
Granulomatous disease			
Age at dx	36 years	22 years	35 years
Disease manifestations	Pulmonary disease	Pulmonary disease, abdominal lymphadenopathy	Erythema nodosum, abdominal lymphadenopathy
Histologic findings	Bronchial epithelioid-cell granulomas	Bronchial and abdominal epithelioid-cell granulomas	Abdominal epithelioid-cell granulomas
Other diagnoses	Sjögren’s syndrome, collagenous colitis	Warm antibody hemolytic anaemia, autoimmune hepatitis, CIDP, progressive spastic paraparesis	

dx, diagnosis; Tg, transglutaminse; AIE-75, autoimmune enteropathy related antigen, 75 kDa; CIDP, chronic inflammatory demyelinating polyneuropathy

### Patient A

A 54-year-old Caucasian woman presented with a clinical symptomatology suggestive for autoimmunity at the age of 32, when she presented with Sicca syndrome and arthralgias. A positive Schirmer’s test, a lip biopsy revealing B and T cell infiltrates, and positive autoantibodies including SS-A(Ro) and SS-B(La) supported the diagnosis of Sjögren’s syndrome.

One year later, CD was suspected since the patient was suffering from chronic diarrhoea and weight loss, but unfortunately no further diagnostic measures were taken. At the age of 41 duodenal biopsies revealed total villous atrophy (Marsh IIIc) and GFD was introduced, which initially resulted in a significant improvement of symptoms. However, during the following 17 years she complained about remitting episodes of diarrhoea. Duodenal biopsies repeatedly confirmed villous atrophy, hence leading to the suspicion of RCD and the referral to our centre. Thus, at 50 years of age, the patient presented to our clinic. At this time transglutaminase (Tg)-IgA antibodies were positive, duodenal mucosa showed intraepithelial lymphocytosis without crypt hyperplasia or villous atrophy (Marsh I). After optimizing GFD, symptoms ameliorated and Tg-IgA turned negative, altogether suggesting incomplete GFD as the underlying cause for villous atrophy. When the patient presented again with diarrhoea at the age of 53 the duodenal mucosa appeared normal (Marsh 0) and Tg-IgA were negative. Duodenal IEL revealed a normal phenotype and an oligoclonal pattern. Colonic biopsies displayed a thickened collagen band compatible with the diagnosis of collagenous colitis.

Sarcoidosis was diagnosed at the age of 36 when a chest radiograph incidentally revealed intrathoracic lymph node enlargement. Consecutive bronchial biopsies revealed an atrophic mucosa containing epithelioid-cell granulomas without central necrosis as they are typically found in sarcoidosis. Staining for acid-fast bacilli was negative. The CD4/CD8 ratio of bronchial lymphocytes was 0.8 and the patient received a course of prednisolone (initially 40 mg qd that were tapered over a 6 months time period). 18 years later she presented with fatigue and an elevated erythrocyte sedimentation rate (1st hour: 78 mm). A CT-scan of the chest revealed bi-pulmonic interlobular septal thickening and ground-glass opacities. Bronchial mucosa appeared inflamed. Again, no evidence of mycobacterial infection was present as interferon-γ release assay, staining for acid-fast bacilli, and mycobacterial cultures from broncho-alveolar lavage all turned out to be negative. Serum soluble interleukin-2 receptor (sIL-2r) was elevated, hypercalcaemia was not present. At the same time extractable nuclear antigen antibodies, SS-A(Ro), SS-B(La), and gamma globulins were increased. HLA-typing revealed the DR3/DQ2 alleles (Table 2).

**Table 2:** HLA status

Patient	A	B	C
HLA class I	A1/A68	n.d.	n.d. except for negativity of B27
	B7/B8		
	C2/C7		
HLA class II	DR3/DR15	DR9/DR15	DR3/DR16
	DQ2	DQ6/DQ9	DQ2/DQ5

n.d., not done

### Patient B

The 59-year-old Caucasian male presented first at the age of 22 with dyspnoea on exertion and radiographic signs of pulmonary disease. Bronchial biopsies indicated epithelioid-cell granulomas and sarcoidosis was diagnosed. During the following decades his disease progressed to stage IV pulmonary sarcoidosis with bi-pulmonic fibrosis and bi-hilar lymphadenopathy accompanied by diffuse abdominal lymphadenopathy. Pulmonary mycobacteriosis was excluded repeatedly by negative staining for acid-fast bacilli, negative polymerase chain reaction for *M. tuberculosis* DNA, and negative mycobacterial cultures of broncho-alveolar lavage material. Furthermore, no evidence was found for fungal infection or presence of anorganic particles as an underlying cause of pulmonic granuloma formation. Lymph node extirpations from the groin and the hepatoduodenal ligament revealed epithelioid-cell granulomas. Serum levels of sIL-2r, calcium, 1,25-dihydroxy-vitamin D, and angiotensin converting enzyme were repeatedly elevated. However, a rheumatologic workup including anti-neutrophil cytoplasmic antibodies turned out negative. He therefore received several courses of steroid treatments.

CD was diagnosed at the age of 34 as the patient suffered from chronic diarrhoea and weight loss. The patient recalled later that at this stage the diagnosis was based solely on the finding of duodenal atrophy but not on serology. For the following 23 years the patient followed a strict GFD, however with relapsing episodes of diarrhoea and weight loss. Repeatedly, duodenal biopsies proved total villous atrophy with crypt hyperplasia and intraepithelial lymphocytosis. At these follow-up visits to our clinic, CD-associated antibodies (Tg-IgA/IgG, endomysium-antibodies, gliadin-IgA/IgG) were analyzed but turned out to be negative. In addition, antigen expression and T-cell receptor gene rearrangement analyses of IEL did not indicate monoclonality.

Secondary to a diagnosis of warm antibody hemolytic anaemia at the age of 47, treatment with steroids was initiated again with maximum doses of up to 150 mg prednisolone qd, which was extended to cyclophosphamide, mycophenolate, and finally splenectomy as well as rituximab. Interestingly, immunosuppressive treatment was paralleled by amelioration of diarrhoea and duodenal mucosal findings. When the patient presented again with diarrhoea at the age of 52, autoimmune enteropathy (AIE) related antigen-75 antibodies were analyzed and found to be significantly elevated. Based on these findings, AIE was diagnosed and treatment with azathioprine initiated. At 53 years of age the patient developed acute autoimmune hepatitis that required escalation of immunosuppressive treatment. Later on, GFD was discontinued without worsening of abdominal symptoms. Six months after the re-introduction of gluten, Tg-IgA remained negative and duodenal mucosa was normal except for a moderate lymphocytosis. Corresponding to the previous findings, HLA-typing revealed that he neither carried the DQ2- or DQ8-haplotype, but the DQ6/DQ9 and DR9/DR15 alleles (Table 2). Taking all criteria together, the patient never had CD.

Beginning at the age of 47, patient B suffered from a complex neurological disorder. He presented with gait abnormalities that corresponded to a slowly progressive spastic paraparesis, obstipation and overflow incontinence. Extensive diagnostics included normal MRI scans of the cranium and spine. Transcranial motor cortex stimulation showed signs of first motor neuron degeneration explaining the spastic paraparesis. Lumbar punctures revealed moderate lymphomonocytic pleocytosis (cell counts ranging from 9-13/μl) and mild disturbances of the blood–brain barrier, but a lack of intrathecal immunoglobulin synthesis. Sensorimotor polyneuropathy was diagnosed by electroneurography (ENG) with slightly reduced nerve conduction velocities and increased F wave latencies. A biopsy of the sural nerve showed demyelination. With multiple previous autoimmune disorders in mind two diagnoses were made: **(i)** a presumably immune-mediated myelopathy being responsible for most of his focal neurological symptoms including spastic paraparesis and **(ii)** chronic inflammatory demyelinating polyneuropathy (CIDP), explaining demyelination, ENG, and liquor results. Lacking an adequate treatment for myelopathy, treatment focussed on CIDP. Thus, he received intravenous immunoglobulins (IvIg). Symptoms did not improve with therapy. However, IvIg application was continued to avoid deterioration of CIDP.

### Patient C

**Fig. 1:**
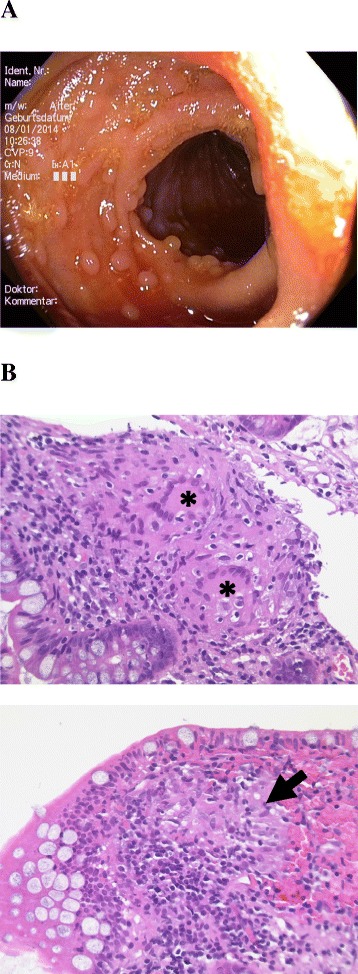
Ileal findings of patient C. **a**) Ileocolonoscopic follicular hyperplasia. **b**) Hematoxylin and eosin staining of ileal biopsies showing multinucleated macrophages (asterisks) and an epithelioid-cell granuloma (black arrow)

Since childhood the 47-year-old Caucasian male was suffering from chronic abdominal discomfort, bloating, diarrhoea, and weight loss. At the age of 35 CD was suspected for the first time when duodenal biopsies revealed total villous atrophy and crypt hyperplasia. Gliadin-antibodies were highly positive. CD was also diagnosed in two of his brothers and in his niece. At the same time a CT-scan of the abdomen revealed iliac, paraaortic, and mesenteric lymphadenopathy as well as splenomegaly. Lymph node analysis showed epithelioid-cell granulomas without any histopathological evidence for mycobacteriosis. The patient started a GFD that led to partial improvement of his symptoms and normalization of duodenal mucosa as well as Tg-, gliadin-, and endomysium-antibodies. However, episodes of abdominal discomfort and diarrhoea remitted. Neither duodenal histology nor flow cytometric analysis of IEL indicated an abnormal IEL phenotype. Repeated colonoscopies performed between the ages of 41 to 46 years revealed a follicular hyperplasia of the terminal ileum without any evidence for mucosal ulcerations as typically found in Crohn’s disease (Fig. 1). Histology of one biopsy from the terminal ileum uncovered epithelioid-cell granulomas. A thorough work-up for an underlying infectious disease was inconclusive, except for positive antibodies against *Yersinia spp*. HLA-typing revealed the carriage of DR3/DQ2 (Table 2).

From the age of 44 onwards, the patient suffered from painful erythema nodosum of both legs accompanied by arthralgias of multiple joints. He also complained about severe fatigue and persistent cough. Chest radiograph, bodyplethysmography, and bronchoscopy were without pathological findings. In particular, bronchoscopy did neither reveal any evidence for granuloma of any type nor for *M. tuberculosis* by microscopy, polymerase chain reaction and mycobacterial culture. A rheumatologic work-up including Rheumatoid factor, anti-nuclear antibodies, anti-neutrophil cytoplasmic antibodies, testing on HLA-B27 and MRI of lumbar spine and sacroiliac joints was not conclusive. Functional testing of immune cells did not support chronic granulomatous disease.

In summary, case C includes the diagnoses CD and a granulomatous disease, partially resembling Löfgren’s syndrome, the latter being not only supported by the record of granulomata found in an enlarged lymph node but also by carriage of HLA-DR3.

## Conclusions

Although the first description of patients with combined diagnoses of CD and sarcoidosis dates back to 1984 [1], clinical evidence of disease association has remained sparse ever since. In a first systematic investigation on intestinal immune reactivity in sarcoidosis increased gliadin-antibodies were present in 41 % of sarcoidosis patients compared to 5 % in controls whereas no differences were observed in antibody levels against casein, β-lactoglobulin, and ovalbumin. Independent of gliadin-antibodies, IEL counts were significantly raised in sarcoidosis patients [14]. Elevated gliadin-antibody levels in sarcoidosis patients were found in another study, but no alterations in IEL counts or villous atrophy were observed [4]. Retrospectively, one might critically assume that gliadin serology can be false-positive at high rates in patients in whom other autoimmune disorders are already known [15].

However, a case–control study investigating the prevalence of CD amongst patients with sarcoidosis reported a trend to a higher prevalence (4 % in sarcoidosis patients when compared to 1 % in controls). Interestingly, 80 % of sarcoidosis patients being additionally diagnosed with CD were HLA-DR3/DQ2 carriers, suggesting a genetic link between both diseases [5].

Inversely, a case control study investigating 335 CD patients identified five cases of sarcoidosis compared to none in the control group [16]. Most recently, a data-based review of 866 biopsy proven cases of CD revealed 10 cases of sarcoidosis, hence being 33-fold higher than the expected number in a matching Caucasian American population [6].

In Caucasians, CD and sarcoidosis are often linked to a highly conserved haplotype, called the 8.1 ancestral haplotype. Amongst HLA-DR3/DQ2, it further contains the HLA-A1, −B8, C4A null, and tumor necrosis factor (TNF)-α-308 alleles. It has been associated with numerous autoimmune disorders (including Sjögren’s syndrome, dermatitis herpetiformis, IgA-deficiency or insulin-dependent diabetes mellitus) possibly due to an increased transcription of TNF-α and reduced complement-mediated antigenic clearance [17]. To our knowledge no overlap in non-HLA risk genes has been described between CD and sarcoidosis.

With these reports in mind, we searched for patients with the combined diagnoses of CD and sarcoidosis at our institution and subsequently identified three patients with malabsorptive syndromes secondary to duodenal villous atrophy and a granulomatous disease.

Based on the positive serology, duodenal histology and the normalization of symptoms after introduction of GFD, the diagnosis of CD could be established in patients A and C. Interestingly, both share the HLA-DR3/DQ2 haplotype. Moreover, in patient A, collagenous colitis and Sjögren’s syndrome, diseases likely to be associated with CD, were established [18]. In patient C, the finding of epithelioid-cell granulomas initially raised the suspicion of Crohn’s disease. However, this could not be confirmed, as no other findings supported this diagnosis. In conjunction to lymph node granulomas, frequent erythema nodosum and the genotype, the ileal epithelioid-cell granulomas were interpreted as an intestinal manifestation of sarcoidosis.

Patient B adhered to GFD for 23 years due to the putative diagnosis of CD – however, without convincing relief of malabsorption-associated symptoms. Since celiac-specific serology was negative, GFD did not improve symptoms, HLA-DQ2 and -DQ8 analysis were negative and anti-enterocyte AIE-75 antibodies were found to be positive, AIE was diagnosed and GFD was discontinued. AIE is a rare disease that mainly occurs in children and young adults, but may also affect elderly patients. Proposed diagnostic criteria from the so far largest case series include adult-onset protracted diarrhoea not responsive to any dietary exclusion and associated with intestinal villous atrophy, circulating gut autoantibodies (e.g. against enterocytes or goblet cells), and/or predisposition to autoimmunity. About 60 % of the adult cases presented with abdominal lymphadenopathy on CT, a finding also observed in patient B. AIE tends to be unremitting without aggressive immunosuppression but complete or partial remission can be achieved in about 60-80 % of patients [19]. Normalisation of the duodenal mucosa despite discontinuation of GFD as observed in this patient might be due to the immunosuppression he received for his other autoimmune diseases.

Patients A and B displayed findings in line with the diagnosis of sarcoidosis including clinical symptoms, radiologic evidence of pulmonary disease, and epithelioid-cell granulomas. However, two findings of patient A argue against sarcoidosis. First, the comorbid diagnosis of Sjögren’s syndrome. Estimations of lung involvement in Sjögren’s syndrome vary widely from 7-75 % with nonspecific interstitial pneumonitis being the most common form of interstitial disease manifestation. Although recent evidence suggests the coexistence of sarcoidosis and Sjögren’s syndrome in the same individual and even argues for a true association [20], the American-European study group on classification criteria for Sjögren’s syndrome stated in 2002 both diseases would exclude each other [21]. Second, patient A’s bronchial CD4/CD8 ratio was <3.5, a finding with a negative predictive value of 85 % for the diagnosis of sarcoidosis [12]. In this case, the interdisciplinary inflammation team at our centre continued to work with a diagnosis of sarcoidosis since it best explained the granulomatous disease.

Besides CD, patient C suffered from a chronic granulomatous disease including abdominal lymphadenopathy with proven epithelioid-cell granulomas and severe erythema nodosum. His symptoms included fatigue, arthralgias, and persistent cough. Pulmonary findings were normal. Repeated examinations did not reveal further evidence for Crohn’s disease, a particular rheumatologic or infectious disease. Sarcoidosis is a diagnosis of exclusion. However, without hilar lymphadenopathy the diagnosis should be made cautiously. In the so far largest case control series of sarcoidosis, 8 % of patients had a normal chest radiograph and 2 % had only extra-thoracic disease [22]. Technically, the clinical presentation together with epithelioid-cell granulomas and the lack of clear evidence supporting alternative diagnoses fulfil the diagnostic criteria of extra-thoracic sarcoidosis.

Patients B represents to our knowledge the first case reported so far with the combined diagnoses of AIE and sarcoidosis. It also illustrates that HLA typing might serve to exclude CD. The case further exemplifies how difficult it might be to define exact diagnoses in patients with complex autoimmune diseases, a fact that may bias association studies.

A large body of evidence links distinct HLA variants to either sarcoidosis or CD. In the latter, the HLA-DQ locus appears to have the biggest impact on disease development with the majority of patients carrying a variant of HLA-DQ2 (DQA1*05/DQB1*02) and only 5 % carrying HLA-DQ8 [23]. On the other hand, the HLA-DR3, −DR11, −DR12, −DR14, and -DR15 alleles are established risk factors for sarcoidosis with the HLA-DR3 haplotype being typically associated with Löfgren’s syndrome [24]. Evidence from clinical and genetic studies support an association of CD and sarcoidosis most likely due to linkage disequilibrium of HLA-DQ2 and -DR3. Patients A and C may exemplify this. A similar mechanism appears to underlie the association of CD with other autoimmune diseases that are linked to the common ancestral haplotype 8.1.

In summary, this case series exemplifies rare coincidences of granulomatous inflammations/sarcoidosis and CD/autoimmune enteropathy. These cases as well as others described in the literature underline the relevance of a distinct genetic HLA configuration for the development of these diseases. Besides, the case of patient B suggests that the diagnosis of CD should be reconsidered in patients without persuading improvement to GFD. In such peculiar cases different diagnostic methods may be required to rule out RCD including HLA-typing as well as antigen expression and T-cell receptor gene rearrangement analyses of IEL. In cases with retrospectively low probability for CD, GFD should be discontinued in favour of quality of life. However, consecutive control examinations should exclude deterioration thereafter.

## Abbreviations

AIE: Autoimmune enteropathy
CD: Celiac disease
GFD: Gluten-free diet
HLA: Human leukocyte antigen
IEL: Intraepithelial lymphocytes
RCD: Refractory celiac disease
sIL-2r: Soluble interleukin-2 receptor
Tg: Transglutaminase
TNF: Tumor necrosis factor
